# A Case of False Aneurism of the Brachial Artery, on Which an Action Was Brought for Malpractice, with Recovery of Damages

**Published:** 1846-08

**Authors:** E. D. Foree

**Affiliations:** New Castle, Ky.


Art. III.—*A Case of False Aneurism of the Brachial Artery, on which an action was brought for Malpractice, with recovery of damages.* Reported by F. D. Foree, M.D., of New Castle, Ky.

At the July Term (1844) of the Henry Circuit Court, New Castle, Kentucky, Hon. Judge Pryor presiding, Boon Regland, through his attorneys, Wilson & Buckley, complained, that Dr. Geohaghan had negligently, carelessly, or unskilfully wounded an artery at the bend of the right arm, which had caused a false aneurism, thereby producing to him much pain, long suffering, and a partial loss of the use of his arm, making it necessary for him to expend much money in procuring its restoration; upon which plea he claimed of defendant, in money, a fair remuneration for the injury.

The defendant, Dr. Geohaghan, through his attorneys, Nuttall, Webb, and McHenry, plead not guilty, whereupon the following proof was presented to the jury:

Mr. Ferris, student of defendant, testified, that Dr. Geohaghan is a practising physician of this county; that about the 12th of last November the plaintiff came to defendant's office and asked witness if he could bleed, adding that his physician, Dr. Goslee, had directed him to lose blood. Witness replied that he could, but being engaged, defendant rose from the bed upon which he was resting, corded plaintiff's right arm, and bled him. The blood was venous in its appearance and flowed in a large regular stream, without any jetting impetuosity; the orifice was dressed in the usual mode; the flow of blood was easily arrested; patient complained of faintness, but of nothing else, and left defendant's office after the lapse of a few minutes. The second day afterwards he returned; the arm was now much swollen, and straw-colored; plaintiff complained of its being very painful, whereupon a poultice was directed by defendant. A week or ten days subsequently, witness, at the request of defendant, went to plaintiff and solicited him not to submit to amputation which he understood had been advised, proposing to take him to Dr. Dudley, in Lexington, to be treated by him, and to defray all the expenses that might be incurred by the trip.

Mr. Wells testified, that he accompanied plaintiff to defendant's office; saw the latter perform the venesection; thought defendant made the incision very hurriedly; used the thumb lancet; held it between the thumb and index finger; did not rest his hand upon plaintiff's arm.

Mr. Scott affirmed, that he saw plaintiff about two hours after he was bled; he then complained of great feebleness, and of intense pain in the arm, which was considerably swollen about the elbow; saw patient frequently during his long confinement; saw him two or three days after he was bled, when his arm was excessively swollen, the tumor extending to the shoulder in one direction and to the fingers in the other. The limb was of a straw color and very painful. Witness told defendant that he was instructed by plaintiff to say to him, that he would freely forgive him the injury done if he would visit him, (the plaintiff). Defendant did not comply with plaintiff's request.

Dr. Goslee testified, that plaintiff called on him at his office in this town on the 22d November. Witness invited his friend, Dr. Owens, to see the case with him. Patient told him that ten days previously he had been bled; that the arm swelled very much a few hours afterwards, and was very painful; that the second day after the bleeding he had applied poultices, and had continued them until that time. The limb was excessively swollen and indurated throughout its whole length; was not very tender to the touch; of a straw color, not very painful, but quite hot; had at the wrist a very small, quivering, and thread-like pulse. Witness could not make out a very satisfactory diagnosis, but thought it might be phlebitis; patient had come about eight miles on horseback to see him, and returned to his home the same day. He directed the patient to continue the poultices and wash the arm with a solution of sugar of lead. On the 24th of the same month he visited him at his father's house; found the limb not so much swollen, œdematous, and less painful, applied a bandage which was directed to be kept wet with a solution of sugar of lead; gave a purgative.

29th. Visited him again; found the limb not so much swollen, and its surface cool. Directed rubefacient frictions, and the bandage to be continued.

From that time until the 24th of the succeeding month he visited patient frequently; did not vary the treatment materially, and but little alteration occurred in the state of the limb. Meanwhile, patient's general health rapidly declined. On unrolling the limb, Dec. 25th, coagulated blood passed out through the original orifice, where an ulcer had now made its appearance. Witness then became satisfied that it was aneurism, and sent patient to a surgeon in Louisville to be treated by an operation.

Dr. Drane testified, that on the 26th of December plaintiff called upon him, in Louisville, to examine his arm. Witness believed it to be aneurism caused by injury of the artery. On the following morning, assisted by Dr. Gross, he cut down and tied the brachial artery above and below a wound which they found in it, and afterwards pressed out through the incision 25 or 30 ounces of blood, which had been effused into the cellular tissue of the arm. The disorganization of the parts was so great that witness could not ascertain precisely the nature and extent of the opening in the artery, but, judging from the size of the stream of blood which jetted out, it must have been a large one. Patient was much emaciated, and so enfeebled that that he could not stand or sit upright; had no pulse in radial artery; remained in the care of witness three months, when he was discharged nearly convalescent. The charges of witness and Dr. Gross for medical attendance on plaintiff were $150; the other expenses incident to his stay in the city were not less than $50. Dr. Drane on being asked, said he believed the defendant to be a well-informed physician, though has no personal acquaintance with him; thinks such accidents must always occur as the result of carelessness, or unskilfulness; they have happened to distinguished surgeons, but never ought to occur; with care can always be avoided. A vein may be transfixed, and an artery wounded, and yet by a change of relative position of the two vessels the arterial bleeding not be manifest.

Dr. Wright testified, that he had known defendant several years; believes him to be a good physician, and a dexterous surgical operator.

After several able speeches by the attorneys, the case was submitted to the jury; after an absence of half an hour they brought in a verdict of $275 for the plaintiff which was confirmed by the Court.

The surgical interest of the foregoing case is not inconsiderable. In it we are presented with evidences of a simultaneous transfixing of a vein and wounding of an artery, without any of the phenomena usually resulting from such accidents.; the flow of blood, *per saltum*, the early formation of a defined tumor, and escape of arterial blood, all being absent; nor was there any pulsation, either at first or subsequently, in the swelling, or any symptom, or physical state of the limb which led either of the physicians first in attendance to suspect the existence of aneurism until the forty-third day after the reception of the injury; when, blood having escaped through the ulcerated orifice, aneurism was suspected. Moreover, Dr. Goslee testifies, that pulsation in the arteries of the wrist was present at his first examination and on several of his succeeding visits, thus still further embarrassing the case, and showing how very difficult it occasionally is to make out a satisfactory diagnosis from the symptoms and physical appearances present at the time, without a faithful and intelligent history of the whole case. So ambiguous were the symptoms here, that, even after the arrival of the patient in Louisville, a distinguished surgeon who saw him pronounced that the tumor was not an aneurism, but a diffused abscess in the cellular substance; nor could he be convinced until an incision was made into the parts and the blood brought to view.

The case is interesting in another point of view. It shows the law rigorous and prompt in imposing penalties for the careless or the unskilful practice of surgery, while it makes no provision for the attainment of surgical knowledge. It exhibits the Commonwealth as punishing its citizens for not understanding anatomy, while it punishes them if they are detected in the study of anatomy. A surgeon is fined, if, in bleeding a patient, his lancet chances to go too deep and pierce an artery lying in its range; and he may be fined, also, if convicted of having dissected other human bodies than those of criminals for the purpose of learning where the arteries are. Truly, whatever may be said of the "majesty" of the law, in this regard much cannot be said in favor of its consistency.

June, 1846.

